# The Neuronal and Peripheral Expressed Membrane-Bound UNC93A Respond to Nutrient Availability in Mice

**DOI:** 10.3389/fnmol.2017.00351

**Published:** 2017-10-31

**Authors:** Mikaela M. Ceder, Emilia Lekholm, Sofie V. Hellsten, Emelie Perland, Robert Fredriksson

**Affiliations:** Molecular Neuropharmacology, Department of Pharmaceutical Biosciences, Uppsala University, Uppsala, Sweden

**Keywords:** UNC93A, SLC, MFS, MFSD, transporter protein, starvation

## Abstract

Many transporters such as the solute carriers belonging to the Major facilitator superfamily Pfam clan are orphans in that their tissue and cellular localization as well as substrate profile and function are still unknown. Here we have characterized the putative solute carrier UNC93A. We aimed to investigate the expression profile on both protein and mRNA level of UNC93A in mouse since it has not been clarified. UNC93A staining was found in cortex, hippocampus and cerebellum. It was found to be expressed in many neurons, but not all, with staining located in close proximity to the plasma membrane. Furthermore, we aimed to extend the starvation data available for *Unc93a* in hypothalamic cell cultures from mouse. We investigated the *Unc93a* alterations with focus on amino acid deprivation in embryonic cortex cells from mice as well as 24 h starvation in adult male mice and compared it to recently studied putative and known solute carriers. *Unc93a* expression was found both in the brain and peripheral organs, in low to moderate levels in the adult mice and was affected by amino acid deprivation in embryonic cortex cultures and starvation in *in vivo* samples. In conclusion, the membrane-bound UNC93A is expressed in both the brain and peripheral tissues and responds to nutrient availability in mice.

## Introduction

Membrane proteins make up one third of all proteins (Almen et al., 2009) and are of critical importance to nearly every aspect of cell physiology. Transporters are one group of membrane proteins that allows movement of molecules over lipid barriers making it possible for absorption, distribution, metabolism, and excretion (Hediger et al., 2013; Yan, 2015). Transporters of the solute carrier (SLC) family are one group of proteins underrepresented in research (Cesar-Razquin et al., 2015) and numerous transporters remain orphans where details about cellular location and/or function are missing. Traditionally transporters are classified into families based on structure and/or function (Schlessinger et al., 2013; Perland and Fredriksson, 2017) and the largest transporter family in humans is the SLC, comprising approximately 450 members currently divided into 52 subfamilies (Hediger et al., 2013; Cesar-Razquin et al., 2015; Perland and Fredriksson, 2017).

Recently, protein sequence analysis (Perland and Fredriksson, 2017; Perland et al., 2017a) found that the orphan membrane-bound protein UNC93A is closely related to SLCs belonging to the Major Facilitator superfamily (MFS) Pfam clan (CL0015), thereby classifying it as a putative SLC (Perland and Fredriksson, 2017). MFS is a large group of facilitators and secondary active transporters with a diverse substrate profile, function and expression patterns (Pao et al., 1998; Reddy et al., 2012; Yan, 2015). MFS members are significant for vital cellular physiology in both health and disease and it is therefore important to understand their role. The MFS members are present in several biological kingdoms (Hoglund et al., 2011), and UNC93A is conserved in insects, nematodes, rodents, and humans (Levin and Horvitz, 1992; Campbell et al., 2011; Son et al., 2015; Perland et al., 2017a).

Unc-93 was first identified in a genome-wide screening in *Caenorhabditis elegans*, in which it is suggested to be a potassium channel regulatory protein, one of five genes identified to be involved in muscle coordination (Levin and Horvitz, 1992; de la Cruz et al., 2003). In mammals, there are two UNC93 proteins, UNC93A and UNC93B1 (Perland and Fredriksson, 2017). So far there is little research on expression profile, localization and function of UNC93A, while UNC93B1 is reported to be a toll-like receptors (TLRs) signaling regulator, important for the trafficking of TLRs from the endoplasmic reticulum to endolysosomes (Lee et al., 2013). UNC93B1 is also important for TLR5 expression and function at the plasma membrane (Huh et al., 2014). There have been attempts to study the role of UNC93A in humans. Liu et al. (2002) studied an allele loss on chromosome 6 linked to sporadic ovarian cancers that indicated the presence of a putative tumor suppressor gene, where UNC93A was identified within the interval of the allele loss. They continued to evaluate UNC93A as the potential tumor suppressor gene, but Liu et al. (2002) concluded that UNC93A was plasma membrane bound but was not the tumor suppressor gene they searched for. *UNC93A* was also mentioned in a family based association study, as a candidate for pulmonary function in a northeast Asian population (Son et al., 2015) but exactly how *UNC93A* affects the pulmonary function is still unclear. They focused on conserved endosomal pathway proteins during infection of *Aedes aegypti* and found that viral RNA accumulates in UNC93A-silenced mosquitoes (Campbell et al., 2011). However, no further studies on mammals have found involvement of UNC93A in immunity.

Furthermore, our group presented microarray data on mouse hypothalamic N25/2 cell lines subjected to amino acid starvation. Alterations in gene expression of several putative SLCs, including *Unc93a* (Perland et al., 2017a), as well as known SLCs belonging to the SLC2, SLC6, SLC7, SLC16, SLC38, and SLC40 families (Hellsten et al., 2017b). Several of the SLCs belonging to the SLC2, SLC6, and SLC38 family are known to be regulated by nutrient availability (Wertheimer et al., 1991; Nagamatsu et al., 1994; Kilberg et al., 2005; Drgonova et al., 2013) and it is reported that *Slc38a2* contain amino acid response elements (AAREs) and is activated by transcription factor 4 (ATF4), which contribute to the amino acid dependent regulation observed for this particular transporter (Palii et al., 2004, 2006; Tanaka et al., 2005).

We aimed to investigate the expression profile on both protein and mRNA level of UNC93A since it has not been clarified. In addition, we also wanted to extend the starvation data already available for *Unc93a.* We have characterized the putative SLC UNC93A in mice, with focus on protein localization in the central nervous system and mRNA expression changes during different nutrition conditions. Using immunohistochemistry (IHC), we found UNC93A staining in cortex, hippocampus, part of the hypothalamus, but only observed in the fluorescent IHC, and cerebellum. UNC93A was found to be expressed mostly in neurons with staining located in close proximity to the plasma membrane. We also investigated changes in expression levels of *Unc93a* in response to amino acid deprivation in mouse embryonic cortex cells as well as study this in a mouse model, where mice were subjected to 24 h of starvation. *Unc93a* gene expression was found in brain areas and peripheral tissues in low to moderate levels in the adult mice and *Unc93a* was also found to change expression levels due to both amino acid deprivation in embryonic cortex cultures and after starvation in *in vivo* samples from mice. In addition, we studied the presence of promoter motifs and sensing elements within *Unc93a* and 1000 bases upstream of the transcription start site (TSS), were we found one TATA box and two ATF4 motifs.

## Results

### UNC93A Is Evolutionary Conserved in Animalia and Has 12 Potential Transmembrane Helices

**FIGURE 1:**
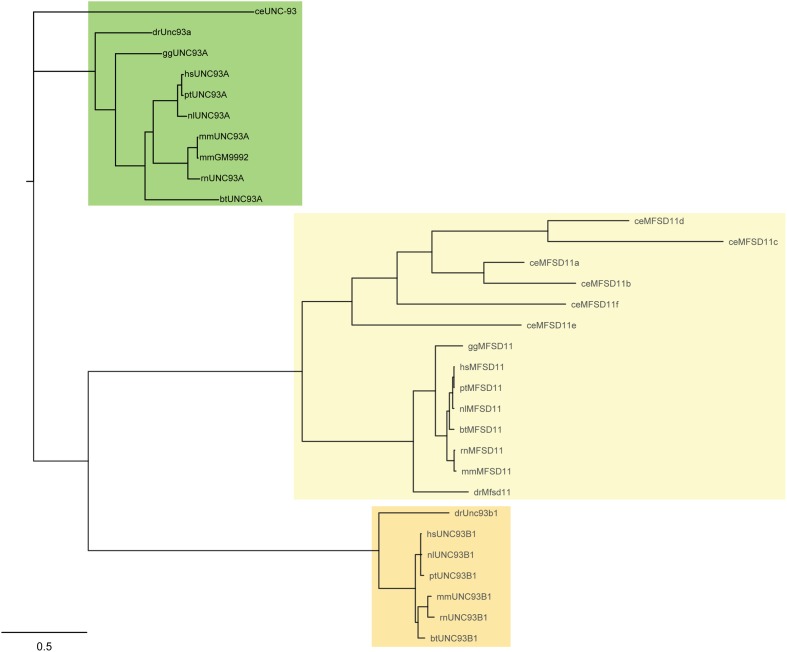
Phylogenetic analysis of UNC93A. Related protein sequences to the human UNC93A were found using a Hidden Markov model (HMM). UNC93A was evolutionary conserved in *Caenorhabditis elegans* (ce), *Danio rerio* (dr), *Gallus gallus* (gg), *Homo sapiens* (hs), *Mus musculus* (mm), *Nomascus leucogenys* (nl), *Pan troglodytes* (pt) and *Rattus norvegius* (rn), and it was closely related to MFSD11 and UNC93B1.

A Hidden Markov model (HMM) for UNC93A was built and used in the search for related sequences to the human UNC93A. The search was carried out in nine different proteomes; *Bos taurus* (bs), *C. elegans* (ce), *Danio rerio* (dr), *Gallus gallus* (gg), *Homo sapiens* (hs), *Mus musculus* (mm), *Nomascus leucogenys* (nl), *Pan troglodytes* (pt), and *Rattus norvegicus* (rn). The model revealed that UNC93A was present in all these species representing several mammalian branches (**Table 1**) and its closest relatives were, MFSD11 and UNC93B1, illustrated by a phylogenetic tree (**Figure 1**). Most similar to hsUNC93A were the orthologs found in *P. troglodytes*, the common chimpanzee, and *N. leucogenys*, the northern white gibbon. In addition, orthologs were present in common laboratory animals such as chicken, mice, and rats, making it possible to study protein expression and function. In mice, there are two orthologs, mmUNC93A and mmGM9992. Using EMBOSS Needle (Li et al., 2015) protein alignments were performed to study protein similarities. Both proteins are similar to each other (99.6%) and to hsUNC93A, 71.9 and 72.1%, respectively.

**Table 1:** Related proteins identified using a Hidden Markov model for mammalian UNC93A.

Species	UNC93A		UNC93B1		MFSD11	
			
	Annotated name	UniProt ID	Annotated name	UniProt ID	Annotated name	UniProt ID
*B. taurus*	btUNC93A	F1MB34	btUNC93B1	E1BBH4	btMFSD11	F1MGS4
*C. elegans*	ceUNC93	Q93380			ceMFSD11a	Q21545
					ceMFSD11b	Q9XV34
					ceMFSD11c	Q19933
					ceMFSD11d	I7LFE4
					ceMFSD11f	Q17824
					ceMFSD11g	Q18251
*D. rerio*	drUNC93A	A0A0A0MPU5	drUNC93B1	F1QYA8	drMFSD11	A0A0R4IKA7
*G. gallus*	ggUNC93A	F1NVP3			ggMFSD11	E1BSA8
*H. sapiens*	hsUNC93A	Q86WB7	hsUNC93B1	Q9H1C4	hsMFSD11	K7EQL2
*M. musculus*	mmUNC93A	Q710D3	mm UNC93B1	E9PYK0	mmMFSD11	Q8BJ51
	mmGM9992	B2RWK3				
*N. leucogenys*	nlUNC93A	G1RLQ6	nlUNC93B1	G1R3Z9	nlMFSD11	G1QQ74
*P. troglodytes*	ptUNC93A	H2QU16	ptUNC93B1	H2Q497	ptMFSD11	H2R447
*R. norvegicus*	rnUNC93A	M0RA42	rnUNC93B1	D3ZDJ4	rnMFSD11	D3ZEI8


**FIGURE 2:**
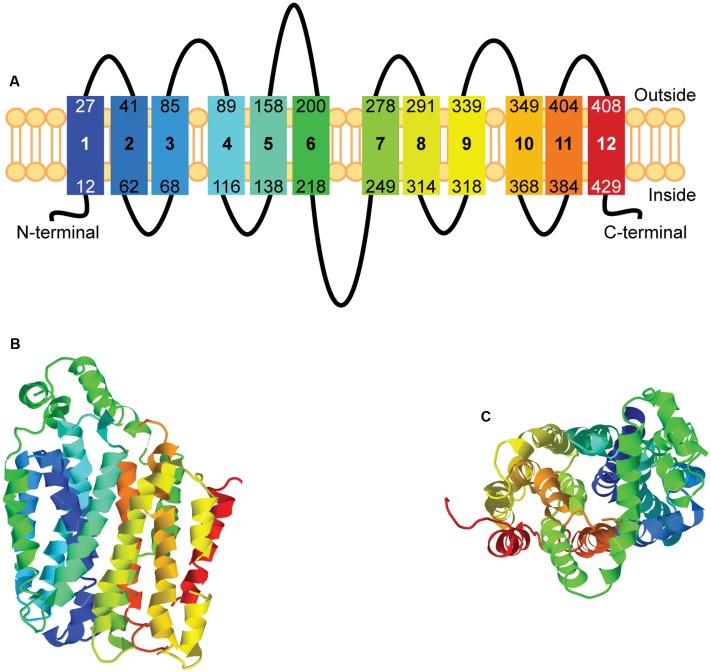
Homology modeling of hsUNC93A. The secondary and tertiary structures of hsUNC93A were modeled using Phyre2. The secondary structure **(A)** of UNC93A revealed 12 transmembrane helices, marked by 1–12, similar to other MFS proteins. The prediction of the tertiary structure illustrated a globular protein (**B**, side-view), with helices packed to form a pore (**C**, top-view). The rainbow gradient of the secondary and tertiary structures shows helices from N-terminal (dark blue) to C-terminal (red).

The secondary and tertiary structures of hsUNC93A were modeled using Phyre2 (Kelley et al., 2015). Homology modeling for UNC93A found a possible structure with twelve transmembrane helices, a common number of transmembrane helices in MFS proteins (Madej et al., 2013), and one alpha helix present in the loop between residue 158 and 200 (**Figure 2A**). Similar to other structure models for putative SLCs (Perland et al., 2017b), UNC93A has the MFS loop present between amino acid 218 and 249. The N-terminus and the C-terminus are shorter (11 and 28 amino acids, respectively) compared to its related protein, UNC93B1 (approximately 60 amino acids or longer) (Perland et al., 2017a), however, the N- and C-termini can vary between SLCs of MFS type (Yan, 2015; Perland et al., 2017a,b). 89% of the residues (407 of the total 457 residues) were modeled with 100% confidence, but only 11% of the amino acid matched the template sequence. However, the high confidence indicates that UNC93A probably adopts the overall fold illustrated, but surface loops can deviate (Kelley et al., 2015). Predictions of the tertiary structure reveal a globular protein (**Figure 2B**, side view), with helices packed to form a pore (**Figure 2C**, top view), reminiscent of other putative SLCs (Perland et al., 2017a,b). The rainbow gradient of the secondary and tertiary structures shows helices from N-terminal (dark blue) to C-terminal (red).

### Verification of Antibodies for Protein Localization Studies

**FIGURE 3:**
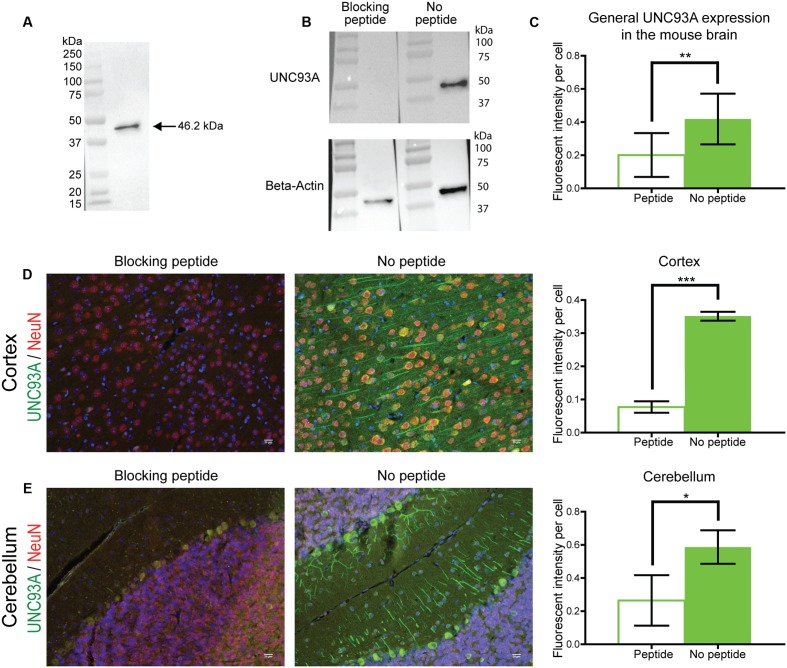
Antibody verification. UNC93A antibody (ab69443, Abcam) used for histological methods was verified using Western blot with a protein samples from HEK293 cells. **(A)** Staining with UNC93A gave one band at 45.2 kDa (expected size at 50.3 kDa). For specificity evaluation an UNC93A peptide (SBP4551, Sigma–Aldrich) that corresponds to the epitope recognized by the antibody was used to block the signal. **(B)** The signal was neutralized in the blocking peptide lane, while a clear band was displayed in the control lane. Beta-actin was run to visualize the amount of loaded protein. Specificity was also tested by performing IHC with and without a blocking peptide on 7 μm brain sections from mouse. The fluorescent intensity was measure using Image J, Fiji edition, and the intensity was corrected to the number of cells in the picture. The graphs represent the average intensity per cell (±*SD*) and expression differences were calculated using *t*-test (^∗^*p* < 0.05, ^∗∗^*p* < 0.01, ^∗∗∗^*p* < 0.001). **(C)** The peptide reduced UNC93A staining in the brain. For example the peptide blocked fluorescent intensity in both **(D)** cortex and **(E)** cerebellum.

Two commercially available antibodies for UNC93A were tested for use in protein localization and were verified using Western Blot on protein samples from HEK293 cells. UNC93A has three known isoforms in human and one known isoform in mice (Cunningham et al., 2015), both orthologs have one N-linked glycosylation site as found by analysis via NetOGlyc 4.0 (Steentoft et al., 2013). Following chemiluminescent detection, one band for the C-terminal anti-UNC93A (ab69443, Abcam) at 46.2 kDa (**Figure 3A**) was seen, with the expected size at 50.3 kDa (MGI:1933250) (Cunningham et al., 2015) (Ensemble release 90). In addition, western blot was performed using C-terminal anti-UNC93A blocked with a peptide corresponding to the epitope recognized by the antibody (**Figure 3B**). The peptide blocked the signal from the C-terminal antibody. Beta-actin confirmed the integrity if the protein sample in both lanes, however, a difference was seen in the amount of loaded protein. However, even if the loaded protein samples amount differed, no sign of UNC93A was obtained in the peptide blocked lane, not even prolonged exposure revealed any staining.

As the antibody was used to localize UNC93A expression in tissue and cell samples, additional verification of the specificity was performed using immunohistochemistry with and without the blocking peptide corresponding to the epitope recognized by the antibody. The peptide reduced UNC93A staining in mouse brain tissue positive for UNC93A (*p* = 0.0088) (**Figure 3C**). For example the peptide blocked fluorescent intensity in both cortex (p =< 0.0001) (**Figure 3D**) and cerebellum (*p* = 0.0379) (**Figure 3E**). The antibody was subsequently used for protein localization analysis as the binding was seen as specific for UNC93A.

For the other available antibody, a N-terminal anti-UNC93A antibody (ab173552, Abcam), a band at ∼52 kDa and at ∼35 kDa (**Supplementary Figure S1A**) were found in a protein homogenate from mouse brain and therefore the antibody was not primarily used for the extensive protein localization studies. Comparative immunocytochemistry on mouse primary cortex cultures did, however, reveal a similar expression pattern as the C-terminal UNC93A antibody (**Supplementary Figure S1B**).

### UNC93A Is Expressed in Cell Bodies and Projections in Cortex, Hippocampus, and Cerebellum of Adult Mice

**FIGURE 4:**
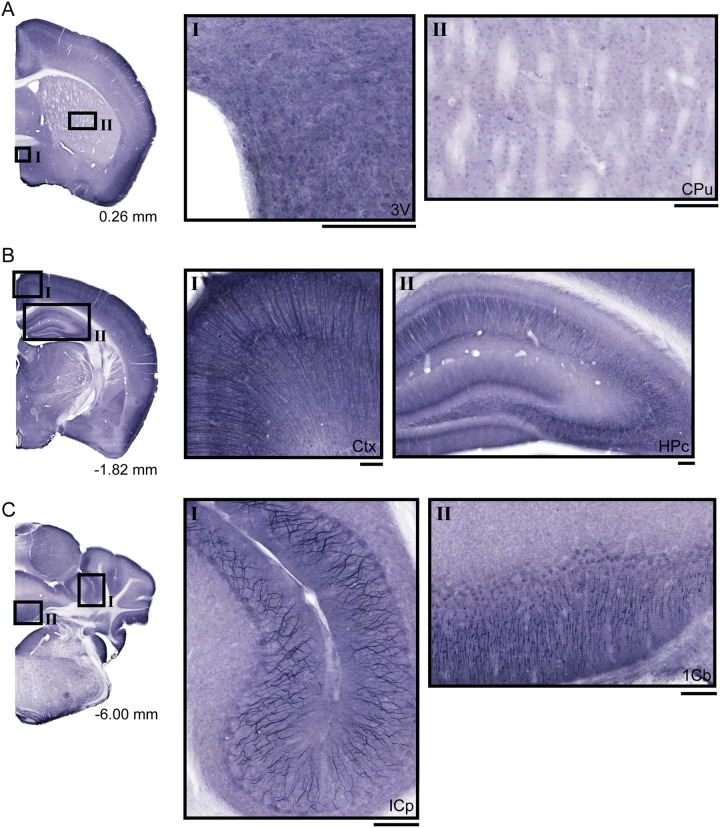
Diaminobenzidine tetrahydrochloride immunohistochemistry of UNC93A in the mouse brain. 70 μm coronal sections from adult mouse brains were stained for UNC93A. **(A)** Brain section from bregma 0.26 mm with close up on **(AI)** areas surrounding the third ventricle (3V) and **(AII)** Caudate putamen (CPu) show no staining. **(B)** In brain sections from bregma -1.82 mm staining was localized to cell bodies and projections in **(BI)** cortex (ctx) and **(BII)** the hippocampus (HPc). **(C)** UNC93A staining was also found in brain section from bregma -6.00 mm. The staining was intense in **(CI)** Purkinje cells in inferior cerebellar peduncles (ICP) and **(CII)** areas in the first cerebellar lobule. The scale bars for the magnifications represent 100 μm.

Non-fluorescent free floating immunohistochemistry on 70 μm thick brain sections were performed for UNC93A to provide an overview of the protein staining pattern throughout the adult mouse brain. Staining was found in cell bodies and projections (**Figure 4**), with especially intense signal in cortex (**Figure 4BI**), hippocampus (**Figure 4BII**) and cerebellum (**Figure 4CI,II**). No staining was seen in the hypothalamic and preoptic areas surrounding the third ventricle (**Figure 4AI**) or striatum (**Figure 4AII**). In cortex, the staining was visual in the majority of the different cortex areas and layers (**Figure 4B**, close up **Figure 4BI**). Long stained projections were detected in the hippocampus (**Figure 4BII**). Furthermore, UNC93A immunoreactivity was also located to the cerebellar lobules (**Figure 4C**), with clear projections of the Purkinje cells both in cerebellar peduncles (**Figure 4CI**) and lobules (**Figure 4CII**).

**FIGURE 5:**
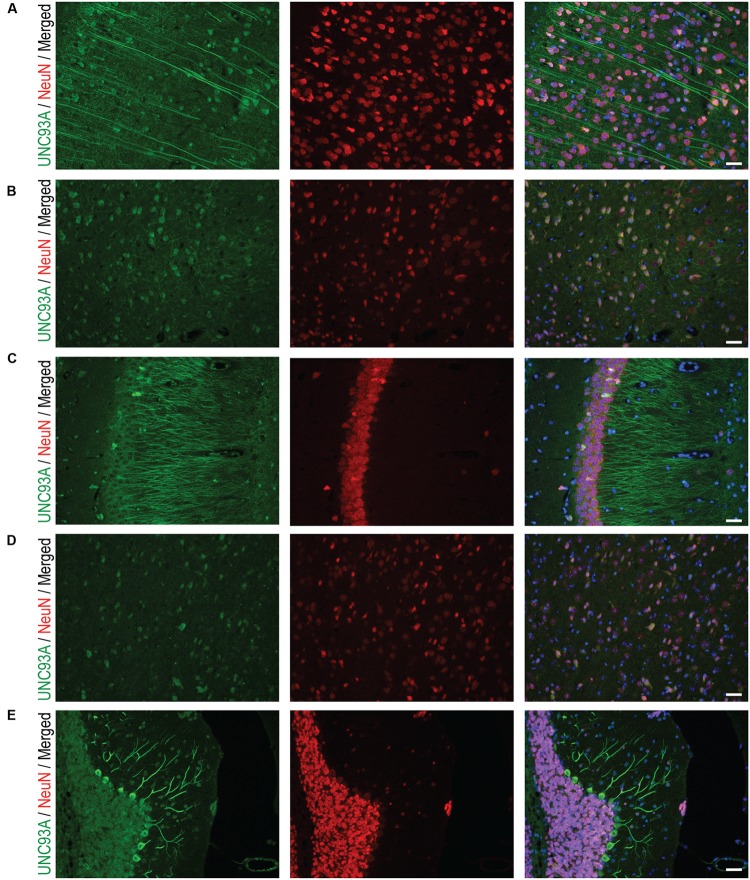
Fluorescent immunohistochemistry of UNC93A and NeuN in the mouse brain. 7 μm paraffin embedded brain sections from adult mouse were stain for UNC93A (green) and NeuN (red) to study the co-localization to neurons. Staining was found in most parts and layers of cortex, e.g., **(A)** motor cortex and **(B)** piriform cortex, as well as **(C)** CA3 in the hippocampus, **(D)** hypothalamus and **(E)** cerebellum. A majority of UNC93A positive cells were also stained with the neuronal marker NeuN, except the Purkinje cells, a GABAergic nerve cell, in cerebellum that does not stain with NeuN. The nuclear marker DAPI (blue) is seen in all merged pictures. Scale bars represent 20 μm.

**FIGURE 6:**
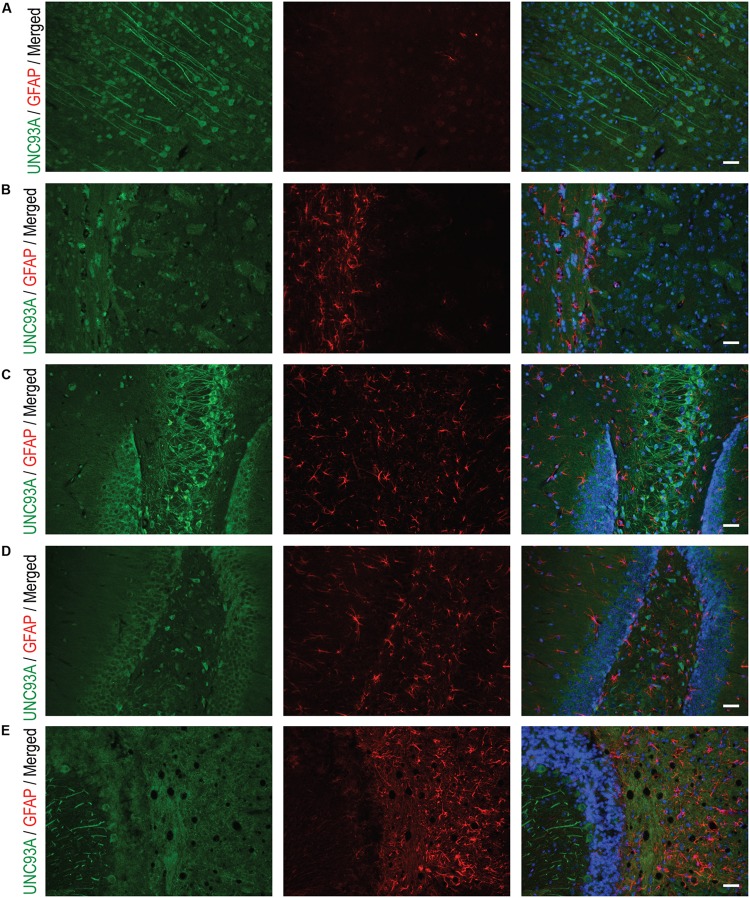
Fluorescent immunohistochemistry of UNC93A and GFAP in the mouse brain. 7 μm paraffin embedded brain sections from adult mouse were stain for UNC93A (green) and GFAP (red) to study the co-localization to astrocytes. UNC93A was found in **(A)** cortex, **(C)** CA3 and **(D)** dentate gyrus of the hippocampus and **(E)** cerebellum. **(B)** No staining was found in striatum. UNC93A did not co-localize to the astrocytic marker GFAP. Nuclear marker DAPI (blue) is seen in all merged pictures. Scale bars represent 20 μm.

To more closely study the localization of UNC93A in brain, fluorescent immunohistochemistry was performed on paraffin brain sections from mice. UNC93A staining co-localized with the neuronal nuclei marker NeuN (**Figure 5**) (Mullen et al., 1992; Sarnat et al., 1998), but not with the astrocytic marker GFAP (**Figure 6**) (Reeves et al., 1989). UNC93A is not present in all NeuN positive cells, indicating UNC93A is only present in a subset of neurons. Signals were also seen in the Purkinje cells, a GABAergic neuron located to cerebellum that will not be stained with NeuN (Gusel’nikova and Korzhevskiy, 2015). Here, as in the non-fluorescent immunohistochemistry, UNC93A staining was detected in cortex (**Figures 5A,B**, **6A**), hippocampus (**Figures 5C**, **6C,D**) and cerebellum (**Figures 5E**, **6E**), as well as in hypothalamic (**Figure 5D**) and no staining detected in striatum (**Figures 5B**, **6B**).

### The Subcellular Localization of UNC93A in Primary Cortex Cells

**FIGURE 7:**
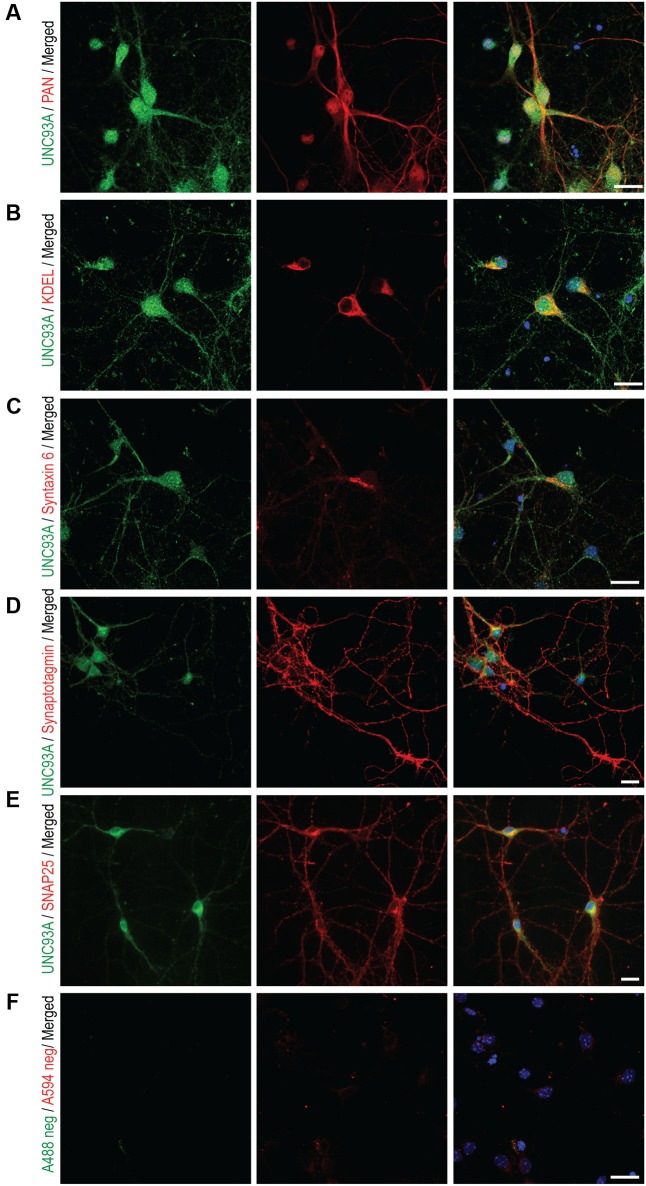
Fluorescent immunocytochemistry of UNC93A in primary cortex cultures. Mouse primary cortex cultures e15 were stained for UNC93A (green) and intracellular markers (red). UNC93A staining (green) combined with **(A)** Pan neuronal staining neurofilament, **(B)** anti-KDEL for ER, **(C)** anti-Syntaxin6 for *trans*-Golgi network, **(D)** anti-synaptotagmin for synaptic vesicles and **(E)** SNAP25 staining the plasma membrane. **(F)** No unspecific staining from the secondary antibodies was seen in the negative control. The nuclear marker DAPI (blue) is in all merged pictures. Scale bars represent 20 μm.

Fluorescent immunocytochemistry were performed on primary cortex cells, prepared from embryos at days 14–16, to determine the subcellular localization of UNC93A. Earlier studies have performed transfection with a clone expressing UNC93A and then concluded that UNC93A is a plasma membrane protein, however, no localization study using antibodies for UNC93A and subcellular parts has been performed previously. Double staining with the Pan Neuronal marker, which stains axons, dendrites, nucleus and cell body of the neurons, confirmed localization of UNC93A to neurons (**Figure 7A**), no co-localization was seen with KDEL (**Figure 7B**), a marker targeting the signal peptide for retention and retrieval of protein to the ER (Raykhel et al., 2007), Syntaxin 6 (**Figure 7C**), targeting the *trans*-Golgi network (Bock et al., 1997), or Synaptotagmin (**Figure 7D**), marking synaptic vesicles (Pevsner et al., 1994). Partial co-localization was observed with SNAP25 (**Figure 7E**), a protein important for vesicle fusion at the plasma membrane (Pevsner et al., 1994). Since the staining of UNC93A was rather modest, a negative control was run to examine the unspecific binding of the secondary antibodies, and no staining was detected for neither Alexa A488 donkey anti-rabbit nor Alexa A594 goat anti-mouse in the absent of primary antibodies (**Figure 7F**). In addition, anti-UNC93A (ab173552, Abcam) was run on primary cortex cells as well to investigate if the second antibody targeting the N-terminal of UNC93A gave similar staining pattern as the C-terminal antibody used for the protein localization studies. The signal for the N-terminal UNC93A antibody correspond well to the C-terminal UNC93A antibody signal (**Supplementary Figure S1B**).

### Unc93a Expression Is Altered after Amino Acid Deprivation in Mouse Primary Cortex Cells

**FIGURE 8:**
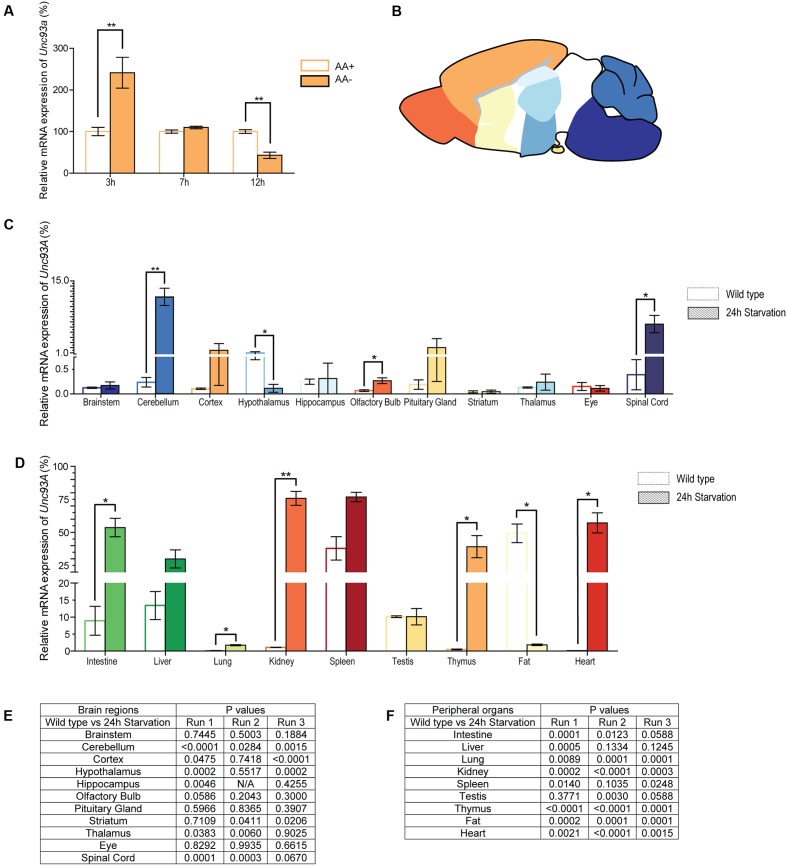
mRNA expression changes of *Unc93a* in cell cultures and tissue samples from mice. The mRNA level of Unc93a in mouse embryonic cortex cultures subjected to amino acid deprivation was monitored after 3, 7, and 12 h. Normalization was performed against three housekeeping genes (*Gapdh, H3a*, and *Actb*) and the relative mRNA expression (±*SD*) was plotted and the gene expression was compared with the controls, set to 100%, for each time point. Expression differences was calculated using *t*-test (^∗^*p* < 0.05, ^∗∗^*p* < 0.01, ^∗∗∗^*p* < 0.001). **(A)** *Unc93a* expression in amino acid deprived cells (AA–, orange) as compared with control cells (AA+, white with orange boarder). Alterations can be seen after 3 and 12 h of amino acid deprivation. **(B)** Picture of mouse brain illustrating the dissected areas used for gene measurement in the adult mice. **(C)** Brain tissue from brainstem, cerebellum, cortex, hypothalamus, hippocampus, olfactory bulb, pituitary gland, striatum, thalamus and eye as well as the spinal cord was taken from mice placed on 24 h starvation (*n* = 5, pooled sample). Normalization was performed to stable housekeeping genes and the qPCR was repeated three times. The merged relative mRNA expression (±*SEM*) was plotted and the expression level monitored in genomic DNA was set to 100% and the normalized quantities were compared to the genomic DNA. Changes were seen in cerebellum, olfactory bulb, spinal cord and hypothalamus. **(D)** Relative mRNA expression of *Unc93a* in peripheral organs. Alterations were observed in intestine, lung, kidney, thymus, heart and fat tissue. Discrepancies were noticed between the three runs, but all runs followed similar mRNA expression pattern regarding expression level and alteration after 24 h hours compared with controls. The *p*-values for all three runs are summarized for **(E)** brain regions and spinal cord as well as **(F)** peripheral organs.

Many SLCs, both putative and known, are involved in metabolism (Nagamatsu et al., 1994; Palii et al., 2004; Hellsten et al., 2017b; Lekholm et al., 2017; Perland et al., 2017b), and the recently amino acid starvation was found to alter *Unc93a* levels in N25/2 hypothalamic cells in mouse (Perland et al., 2017a), the effect of nutrient deprivation on *Unc93a* was studied. Amino acids deprivation induced transient alteration of *Unc93a* expression in primary cortex cells (**Figure 8A**). Gene expression of *Unc93a* was analyzed using qPCR and the regulation was studied in mature embryonic cortex cells cultured in complete or limited amino acid media for 3, 7, and 12 h. After 3 h of amino acid deprivation, *Unc93a* expression was greater than (*p* = 0.0031) the expression in the control samples. After 7 h the gene expression in deprived and control cells were equal, and after 12 h the expression of *Unc93a* was downregulated compared with controls (*p* = 0.0027).

### Gene Expression Changes after Starvation in Adult Mice

As a change in gene regulation was detected in the primary embryonic cortex cultures, the gene expression was also analyzed from brain regions (**Figure 8B**) and peripheral tissue samples from adult mice subjected to 24 h starvation. Gene expression alterations were measured using qPCR and expression was compared with controls. 24 h starvation upregulated the expression of *Unc93a* in cerebellum (*p* = 0.0023), olfactory bulb (*p* = 0.0299) and spinal cord (*p* = 0.0229), while downregulated it in hypothalamus (*p* = 0.0359) compared with controls (**Figure 8C**). In the periphery, the mRNA expression of *Unc93a* was upregulated in intestine (*p* = 0.0319), lung (*p* = 0.0114), kidney (*p* = 0.0049), thymus (*p* = 0.0434) and heart (*p* = 0.0173) and downregulated in fat (*p* = 0.0213) (**Figure 8D**). As the gene expression was normalized to genomic DNA for *Unc93a* for each run, the expression levels can be compared and a clear difference can be seen in the expression between the controls in different tissues.

*Unc93a* had a low mRNA expression profile in the central nervous system, with highest expression in cerebellum, hypothalamus, hippocampus, and spinal cord (**Figure 8C**). In comparison, *Unc93a* was expressed abundantly in peripheral tissues, with the highest mRNA expression in intestine, liver, spleen, testis, and fat (**Figure 8D**).

All qPCR was run three times and the graphs (**Figures 8C,D**) illustrate the merged relative mRNA expression of *Unc93a* and the p-values for all three runs are summarized in **Figures 8E,F**. All three runs followed similar mRNA expression pattern regarding expression level and alteration after 24 h starvation compared with controls.

The response observed for *Unc93a* indicates the presence of transcription factor recognitions motifs important during nutrient deprivation and starvation in the promoter of *Unc93a* and within the gene itself. The whole gene sequence and 1000 bases upstream of the TSS from mouse was investigated, using MEME suite and Eukaryotic promoter database, for promoter motif (CAAT box and TATA box), three different sensing elements: The nutrient-sensing response unit (NSRU), consisting of NRSE-1 and -2 (Zhong et al., 2003), AARE, e.g., ASNS and CHOP that are found in *Slc38a2* (Palii et al., 2004, 2006) and the transcriptional factor ATF4 and CCAAT-enhancer binding protein α (C/EBPα) and β (C/EBPβ) (Averous et al., 2004; Kilberg et al., 2005; Dey et al., 2012). Within the 1000 bases of *Unc93a* was the TATA box, while the CAAT box was not found. Neither the sequence for the NRSU ASNS nor CHOP AARE was found with in the region of interest. However, two copies of ATF4 motifs, approximately 100 and 150 bases upstream of TSS, as well as two copies of C/EBPα motifs, between 740 and 760 bases upstream of TSS, and one C/EBPβ motif, at 740 bases upstream of TSS, were found.

## Materials and Methods

### Identification of Proteins Related to UNC93A and Structural Modeling

A Hidden Markov Model (HMM) was built for mammalian UNC93A proteins using the HMMER package (Eddy, 2011), as described in (Perland et al., 2017b). The HMM was utilized in the search from orthologs to UNC93A by scanning data sets listed in **Table 2**. Proteomes were obtained from Ensembl (Cunningham et al., 2015). The results were manually curated, where splice variants and pseudo genes were removed. The identified entries were combined in a multiple PSI/TM tcoffee sequence alignment (Notredame et al., 2000), before establishing their phylogenetic relations according to the Bayesian approach (Huelsenbeck et al., 2001). For detailed description, see (Perland et al., 2017b). In short, the analysis was run via the Beagle library (Ayres et al., 2012) on five heated and one cold chain. It was performed with two runs in parallel (*n* runs = 2), for a maximum of 2,000,000 generations.

**Table 2:** Data sets retrieved from Ensembl (Cunningham et al., 2015) (release 90).

Species	Common name	Data set
*B. taurus*	Cow	Bos_taurus.UMD3.1.pep.all.
*C. elegans*	Roundworm	Caneorhabditis_elegans.WBcell1235.pep.all
*D. rerio*	Zebrafish	Danio_rerio.GRCz10.pep.all
*G. gallus*	Chicken	Gallus_gallus.Gallus_gallus-5.0.pep.all
*H. sapiens*	Human	Homo_sapiens.GRCh38.pep.all
*M. musculus*	Mouse	Mus_musculus.GRCm38.pep.all
*N. leucogenys*	Northern white-cheeked gibbon	Nomascus_leucogenys.Nleu1.0.pep.all
*P. troglodytes*	Chimpanzee	Pan_troglodytes.CHIMP2.1.4.pep.all
*R. norvegicus*	Rat	Rattus_norvegicus.Rnor_6.0.pep.all


Models of the human UNC93A structure was predicted using Phyre2 (Kelley et al., 2015). The UNC93A structure was modeled against the template with highest confidence, sequence coverage and identity. The glucose transporter from the bacteria *Staphylococcus epidermidis* (PDB id c4ldsB) (Iancu et al., 2013), with 100% confidence and 89% amino acids sequence coverage, were used. Phyre2 provided both the secondary and tertiary predictions. Homology models were modified in Jmol: an open-source Java viewer for chemical structures in three dimensions^1^ (Hanson et al., 2013).

### Animals

The study was approved by the Animal Ethics Committee in Uppsala, Sweden (Permit Number C419/12, C39/16, and C67/13) and is in accordance with the guidelines of European Communities Council Directive (2010/63). Male C57B16/J mice (Taconic M&B, Denmark) were used and considered as wildtype. The mice were housed in an ambient room temperature of 22°C with a 12 h light:dark cycle. All animals were provided food (standard chow R3, Lantmännen, Sweden) and tap water *ad libitum*, unless otherwise stated. All procedures were planned and executed to minimize the suffering and euthanasia was performed during the light period by either transcardiac perfusion or cervical dislocation.

### Western Blot

Total protein concentration on protein samples were analyzed using a Coomassie Protein Quantification Kit (Sigma–Aldrich) and compared to a standard curve of Bovine Serum Albumine (BSA) included in the kit. Western blot was performed as described in (Perland et al., 2016). In short, protein samples, extracted using RIPA buffer (Sigma–Aldrich) supplemented with complete protease inhibitor (Roche) and phosphatase inhibitors (Merck Millipore), from HEK293 cell cultures (1150 μg/ml) or mouse brain tissue (6000 μg/ml) extracted according to (Hellsten et al., 2017a) were diluted 4:1 in Laemmli Sample Buffer (Bio-Rad) with 2-mercaptoethanol (Fluka), and loaded onto a 10% TGX Mini-protean gel (Bio-Rad). Gel separation at 200V for 30 min was performed before blotted onto PVDF membrane using the TurboBlot system (Bio-Rad). As reference a molecular weight marker was used (Prestained dual color, Bio-Rad). The membrane was blocked in 5% milk (Bio-Rad) diluted in TTBS for 1 h before two antibodies for UNC93A was used: Anti-UNC93A (1:100, rabbit, ab69443, Abcam) or Anti-UNC93A (1:80, 1:100, rabbit, ab173552, Abcam) incubation overnight at 4°C. For antibody specificity evaluation an UNC93A peptide that corresponds to the epitope recognized by the antibody was used to block the signal. Here, anti-UNC93A (ab69443, Abcam) was pre-incubated with UNC93A peptide (SBP4551, Sigma–Aldrich) in excess (5:1 to antibody concentration) in room temperature for 1 h prior the incubation. After 3 min × 10 min wash with TTBS, the membrane was incubated at room temperature for 60 min with HRP coupled secondary antibodies, HRP goat α-rabbit, diluted 1:10000 in milk blocking solution (Invitrogen). The membrane was developed using Clarity Western ECL Substrate (Bio-Rad) and the staining was visualized using a CCD camera (Bio-Rad). The staining was compared with the molecular weight marker using Image Lab software v5.2.1 build 11 (Bio-Rad). After detection for the peptide blocked and control membrane, the amount of protein loaded was quantified using beta-actin. Membranes incubated in Beta-actin (Sigma–Aldrich), diluted 1:10000 in 5% milk block, before 3 min × 10 min wash in TTBS and incubation with secondary antibody, HRP chicken α-mouse, diluted at 1:10000 in milk blocking solution (Invitrogen). The membranes were then again developed.

### Collection and Sectioning of Tissue for Non-fluorescent Immunohistochemistry

Male C57Bl6/J mice (Taconic M&B, Denmark) were anesthetized with an intraperitoneal injection of 0.25 ml pentobarbital sodium (Apoteksbolaget, Sweden). Transcardiac perfusion was performed using phosphate-buffered saline (PBS) (137.0 mM NaCl, 2.70 mM KCl, 8.10 mM Na2HPO4) followed by 4% formaldehyde (HistoLab, Sweden) fixation. The brains were dissected and stored in 4% formaldehyde at 4°C overnight. The next day, the brains were washed twice in Tris-buffered saline (TBS) (0.04 M Trizma HCl, 0.01 M Trizma base, 0.15 M NaCl, pH 7.4) followed by 4% agarose gel (VWR, Germany) embedding. 70 μm coronal brain sections were cut with a vibratome Leica VT 1200 S (Leica Microsystems, Germany).

### Non-fluorescent Immunohistochemistry on Free-Floating Mouse Brain Sections

All chemicals were purchased from Sigma–Aldrich, unless otherwise stated. 70 μm brain sections were washed in TBS 4 min × 8 min before and after 10 min incubation in 10% MeOH, 3% H_2_O_2_ (Merck) in TBS. Sections were incubated in 1% blocking reagent (Roche Diagnostics) for 1 h followed by incubation in anti-UNC93A (ab69443, Abcam) diluted 1:200 in supermix (0.25% gelatin, 0.5% Triton X-100 in TBS) at 4°C overnight. Sections were rinsed in TBS 2 × 1+4 min × 8 min followed by incubation in secondary antibody [biotinylated goat-anti-rabbit IgG (H+L), Vector laboratories] diluted 1:400 in supermix for 1 h at room temperature. Sections were rinsed in TBS 5 min × 8 min before and after incubation in Avidin Biotin complex kit [Reagent A, Reagent B (Vectastain, Vector Laboratories)] diluted 1:800 in supermix for 1 h. Immunostaining was visualized by incubating sections in 0.08% 3.3 Diaminobenzidine tetrahydrochloride (DAB), 0.20% NiCl and 0.035% H_2_O_2_ in TBS followed by 4 min × 5 min TBS washes. Sections were mounted on gelatinized microscope Superfrost^®^ Plus slides (VWR) and dehydrated, 5 min in 70% and 95% EtOH, 10 min in 100% EtOH (Solveco) and 20 min in Xylene. Slides were mounted using DPX Mountant for histology (Sigma–Aldrich) with micro cover slides (Menzel Gläser). Sections were analyzed using a Mirax Pannoramic midi scanner with the Pannoramic Viewer software version 1.15.4 RTM (3dHistech). The brightness of the images was adjusted to 75% before compiling the figure.

### Fluorescent Immunohistochemistry of Paraffin Embedded Mice Brain Sections

Brain tissue for immunohistochemistry was collected as described in Roshanbin et al. (2014). Fluorescent immunohistochemistry on paraffin embedded mouse brain sections was performed as described in (Perland et al., 2016) with antibodies all diluted in supermix for anti-UNC93A (ab69443, Abcam, 1:100), NeuN (Merck Millipore, 1:200) GFAP (Merck Millipore, 1:800) and nucleus marker DAPI (Sigma–Aldrich) diluted 1:15000 in PBS. For peptide blocked control section, UNC93A antibody (1:100) was pre-incubated with the corresponding peptide (SBP4551, Sigma–Aldrich) according to procedure described for western blot. Images were acquired using Olympus microscope BX55 with an Olympus DP73 camera and the cellSens Dimension v1.14 (Olympus) and images were then handled using ImageJ, Fiji edition (Schindelin et al., 2012). The differences in fluorescent intensity between the control sections and peptide blocked sections were quantified using ImageJ, Fiji edition. In short, pictures were taken with the same exposure time and the fluorescent intensity for the image was measured and then corrected against amount of cells. The general UNC93A staining in two sections was measured and the staining in cortex and cerebellum was measured in several pictures covering the whole structure. In the graphs, the fluorescent signal is presented as mean ± SD. For statistical analyses, GraphPad Prism software v 5.02: *T*-tests were performed for gene expression alteration where ^∗^*p* < 0.05, ^∗∗^*p* < 0.01, ^∗∗∗^*p* < 0.001.

### Fluorescent Immunocytochemistry on Mouse Primary Cortex Cultures

Wildtype male and female mice were mated and at e14-15 the females were euthanized with cervical dislocation, the embryos removed and cortex dissected out and primary cultures were set up as previously described in Perland et al. (2016). Immunocytochemistry was performed as described in Perland et al. (2016) with anti-UNC93A diluted 1:100 in supermix blocking solution. Co-staining with neuronal marker Pan diluted 1:200 (MAB2300, Millipore), KDEL markers (ab12223, Abcam) diluted 1:200, Syntaxin 6 (Ab12370, Abcam) diluted 1:100, Synaptotagmin (ab13259, Abcam) diluted 1:200 and SNAP25 (ab25737, Abcam) diluted in 1:100 in supermix blocking solution. Images were acquired at the SciLifeLab BioVis Facility (Uppsala University) using confocal LSM710 SIM (Zeiss) and the Zen black software (Zeiss) or Olympus microscope BX55 with an Olympus DP73 camera and the cellSens Dimension v1.14 (Olympus). Images were then handled using ImageJ, Fiji edition (Schindelin et al., 2012).

In addition, immunocytochemistry for the N-terminal anti-UNC93A antibody (ab173552, Abcam), diluted 1:80 in 5% milk block (Bio-Rad), was performed as described above.

### Amino Acid Deprivation in Primary Cortex Cells and Starvation Experiment in Adult Mice

To evaluate if response to amino acid availability alter gene expression of *Unc93a*, primary embryonic cortex cultures were partial deprived of common amino acids as described in Lekholm et al. (2017). In short, EBSS base media (Gibco) containing 1.0 mM Sodium-Pyruvate, 1% Penicillin-Streptomycin, 2% B27, 4X MEM Vitamin solution (Gibco), 10.9 mM HEPES buffer solution was prepared and amino acids L-arginine, L-cysteine, L-lysine, L-methionine, L-phenylalanine, L-proline, L-threonine, L-tryptophan, and L-tyrosine (Sigma–Aldrich) were added. The control media was made as above but also contained additional 2.0 mM GlutaMax (Gibco) and glycine, L-alanine, L-asparagine, L-histidine, L-isoleucine, L-leucine, L-serine, and L-valine (Sigma–Aldrich). At culture day 10, the normal primary culture media, Neurobasal A was aspirated and the amino acid deprived media and control media was added instead. Hence, the cells were subjected to deprivation of glycine, L-alanine, L-asparagine, L-glutamine, L-histidine, L-isoleucine, L-leucine, L-serine, and L-valine (Sigma–Aldrich). The primary embryonic cortex cells were cultures in control media or deprivation media for 3, 7, and 12 h before total RNA extraction, cDNA synthesis and qRT-PCR were performed as detailed below.

Diet experiments were performed as described in Perland et al. (2016) and material was used here to analyze the gene expression pattern of *Unc93a*. In short, adult male mice were starved for 24 h, while having access to water, before euthanasia and dissection, while control mice were kept on standard chow (5% fat, 21% protein, 51.5% carbohydrates). Central and peripheral organs from four mice were dissected and RNA extraction, cDNA synthesis and qRT-PCR were implemented as detailed below.

### Tissue Sampling, RNA Extraction and cDNA Synthesis

Tissues from wildtype and starved mice were prepared and set up as described in Perland et al. (2016). All tissues were stored in RNA later (Qiagen, Sweden) for 2 h at room temperature before placed at -80°C until total RNA was extracted.

Total RNA was extracted using Absolutely RNA Miniprep kit (Agilent Technologies) for mouse tissue samples or RNeasy Midi kit (Qiagen) for cells samples, according to the manufacturer’s instruction and concentrations were measured using ND-1000 spectrophotometer (NanoDrop Technologies). The cDNA synthesis was performed using the Applied Biosystems High Capacity RNA-to-cDNA kit (Invitrogen) following manufacturer’s recommendations. 2 μg RNA template was used for the reaction and the cDNA was pooled and diluted to 5 ng/μl as described in (REF – MFSD5 and MFSD11 manuscript).

### Primer Design and Quantitative Real-Time PCR (qRT-PCR)

All primers were designed using Beacon Design 8 (Premier Biosoft). *Unc93a* (accession no: NM_199252.2) forward 5′-tccactggtactgacaatct-3′ and reverse 5′-aggaggaagccacaagaa-3′. Reference housekeeping genes: *beta tubulin 4B* (*bTub*) forward 5′-agtgctcctcttctacag-3′, reverse 5′-tatctccgtggtaagtgc-3′, *ribosomal protein L19* (*Rpl19*) forward 5′-aatcgccaatgccaactc-3′, reverse 5′-ggaatggacagtcacagg-3′, *histone cluster 1* (*H3a*) forward 5′-ccttgtgggtctgtttga-3′, reverse 5′-cagttggatgtccttggg-3′, *Peptidylprolyl isomeras A* (*Cyclo*) forward 5′-tttgggaaggtgaaagaagg-3′, reverse 5′-acagaaggaatggtttgatgg-3′ and *actin-related protein 1B* (*Actb*) forward 5′-ccttcttgggtatggaatcctgtg-3′, reverse 5′-cagcactgtgttggcatagagg-3′.

*Unc93a* gene expression and expression alteration were determined using qRT-PCR. Final volume for each reaction was 20 μl containing 3.6 μl 10x DreamTaq Buffer (Thermo Fisher Scientific), 0.2 μl of 25 mM dNTP mix (Invitrogen), 1 μl DMSO, 0.5 μl SYBR Green (1:10000, Invitrogen) in 1xTE buffer (pH 7.8), 0.08 μl DreamTaq polymerase (5 U/μl, Thermo Fisher Scientific), 0.05 μl of forward and reverse primer (100 pmol/μl) and 5 μl cDNA (10 ng/μl) for brain and peripheral tissues or 2 μl cDNA (30 ng/μl) for primary embryonic cortex cells. The volume was adjusted with sterile water. An iCycler real-time detection instrument (Bio-Rad) was used with following settings: initial denaturation for 30 s at 95°C, 55 cycles of 10 s at 95°C, 30 s at 55°C for housekeeping genes and 59° for *Unc93a* amplification and 30 s 72°C. A melting curve was initiated for 81 cycles with 10 s interval, starting at 55°C and the temperature increased 0.5°C per cycle. All qRT-PCR was run three times with samples in triplicates, negative control and genomic DNA (10 ng/ul) were included on each plate.

### Analysis of qRT-PCR and Statistics

All data was collected using MyIQ software (Bio-Rad Laboratories). Primer efficiency was calculated using LinRegPCR software v7.5, followed by Grubbs Outlier test (GraphPad software) to remove outliers before calculations based on primer efficiency. The GeNorm protocol (Vandesompele et al., 2002) was used to determine stable reference genes before calculating the geometric mean of the reference genes, which was then used to normalize the gene expression of *Unc93A*. For primary embryonic cortex cultures the gene expression was compared to the controls, set to 100%, for each time point. For brain regions and peripheral tissues samples the genomic DNA was set to 100% and the normalized quantities were thereafter compared to the genomic DNA. Graphs represent merged values for three runs and are presented as means ± SEM. For statistical analyses, GraphPad Prism software v 5.02: *T*-tests were performed for gene expression alteration where ^∗^*p* < 0.05, ^∗∗^*p* < 0.01, ^∗∗∗^*p* < 0.001.

### Analysis of 1000 Bases Upstream of Transcription Start Site and Unc93a

The presence of promoter, transcription factor and sensing elements motifs for mouse *Unc93a* (Accession number: MGI:1933250) and 1000 bases upstream of the TSS were predicted using the MEME suite (Bailey et al., 2009) and Eukaryotic Promoter Database (Cavin Perier et al., 1998). Two promoter motifs, the TATA box and CAAT box, as well as three sensing elements, the Nutrient-Sensing Response Unit (NSRU), which contain the Nutrient-Sensing Response Elements 1 and 2 (NSRE1 and 2) (Zhong et al., 2003), AARE like ASNS and CHOP AAREs that are found in *Slc38a2* (Palii et al., 2006), and the transcription factor Activating Transcription Factor 4 (ATF4) and CCAAT-enhancer binding protein α (C/EBPα) and β (C/EBPβ), were analyzed.

## Discussion

As mentioned earlier transporter proteins such as solute carriers are underrepresented in research (Cesar-Razquin et al., 2015), however, lately there have been an increase in research regarding novel SLCs (Perland et al., 2016, 2017b; Hellsten et al., 2017a; Lekholm et al., 2017). Proteins containing major facilitator superfamily motifs (e.g., MFSD1-14B, UNC93A, and B1, SV2s), which are phylogenetic related to SLCs of MFS type, are, today, suggested to be putative SLCs (Perland and Fredriksson, 2017; Perland et al., 2017a). Here, we have begun to characterize UNC93A, which is one of the proteins that phylogenetically cluster with SLCs of MFS type (Perland and Fredriksson, 2017), but is not similar enough to any known SLC to be grouped into any of the 52 existing SLC families. In humans, UNC93A was found to be most closely related to UNC93B1, a transporter like protein important for regulation and trafficking of toll-like receptors (Lee et al., 2013; Huh et al., 2014), and to the putative SLC MFSD11 (Perland et al., 2016). A Hidden Markov model (HMM) for UNC93A was built and we search for orthologs present in *C. elegans*, in which the Unc-93 protein was first discovered, and mammalian genomes. We found that UNC93A is evolutionary conserved in mammals and its most closely related proteins were UNC93B1 and MFSD11. In addition, the secondary and tertiary structures were predicted for the human protein. UNC93A was modeled based on a glucose transporter in *S. epidermidis*, and found to have 12TMS, similar to other MFS proteins sharing the same evolutionary origin (Madej et al., 2013; Yan, 2015).

Dahlin et al. (2009) studied the expression of 307 Slc genes in the Allen Brain Atlas (Dahlin et al., 2009). UNC93A was not one of the 307 Slc genes included in the screen, and our panel adds information to the expression pattern of transporters in the mouse brain. Co-localization to neurons was verified as UNC93A localized to many cells stained with a the marker NeuN (Mullen et al., 1992), but not with cells positive for the astrocytic marker GFAP. The expression is localized to the cell body of neurons and the projections in cortex, hippocampus and the Purkinje cells. The strong signal present in Purkinje cells and parts of the hippocampus, which is rich in inhibitory neurons (Wheeler et al., 2015; Hamilton et al., 2017), point to that some UNC93A is located to inhibitory neurons. However, additional verification by co-localization studies with neuronal subtype markers is needed to verify the neuronal type UNC93A is expressed in. Earlier work performed by Liu et al. (2002) reported UNC93A to be a plasma membrane protein. Here the intracellular localization was studied with markers for ER, Golgi, Synaptic vesicles and plasma membrane in embryonic cortex cells. Similar to the staining observed in the adult mouse sections, UNC93A staining was seen in cell bodies and the staining was evenly distributed over the cell (**Figure 7**). Once again the co-localization with the Pan neuronal marker indicated a neuronal localization for UNC93A, but pin-pointing the subcellular localization of UNC93A was challenging. UNC93A did not co-localize to intracellular markers for ER, Golgi or synaptic vesicles. Furthermore, UNC93A only partially co-localized with SNAP25, here used as a membrane marker. Taken together, UNC93A was localized close to the plasma membrane, as reported earlier, however, it was not located to the plasma membrane exclusively and the possibility that UNC93A can be expressed in other types of vesicles or organelles that have not been stained for in this study cannot be ruled out. The partial overlap observed between UNC93A and Pan neuronal marker and SNAP25 antibody, as well as the granular staining of UNC93A, highlights the possibility that UNC93A, similar to UNC93B1 is involved in trafficking of proteins. The ortholog to UNC93A in *C.elegans* is believed to be one out of five genes identified in the screen which regulates muscle contraction (Levin and Horvitz, 1992; de la Cruz et al., 2003), and it is suggested to function as a subunit of a potassium channel, SUP-9 which is similar to the human TWIK related acid-sensitive K^+^ (TASK) channels. These channels are important for resting membrane potential, action potential duration and modulating the responsiveness to synaptic input (Duprat et al., 2007). de la Cruz et al. (2003) reported that SUP-9 was similar to the human TASK-1 and TASK-3 (de la Cruz et al., 2003), but there is also a Tandem Pore domain K+ Channel subunit (Karschin et al., 2001), TASK-5, that is related to SUP-9 found when we searched for homologs using NCBI homologene^2^ or phylomeDB^3^. It is therefore possible that UNC93A together with TASK channels are needed for controlling the potassium flow in neurons. TASK-1 and TASK-3 are widespread in the rat brain, but no expression was detected in, e.g., the Purkinje cells (Karschin et al., 2001; Medhurst et al., 2001; Marinc et al., 2014), where we observe extensive UNC93A staining. The expression pattern of TASK-5 in the mouse brain is low with expression located mostly to the auditory system, olfactory bulb mitral cells and the Purkinje cells (Karschin et al., 2001). The differences in protein expression patterns for UNC93A and the TASK channels point to the possibility that UNC93A is not linked to the TASK channels.

Nutrients (glucose, amino acids, and lipids) are simple compounds needed to produce energy and cellular biomass and, due to their importance there are distinct mechanisms to sense intracellular and environmental levels (Efeyan et al., 2015). During starvation both levels of nutrients and ion transport are reduced (Hamwi et al., 1967; Zhao and Willis, 1988). This initiate integrated biochemical and physiological changes that affects not only organs involved in absorption, distribution, metabolism and excretion (ADME) but the whole body energy requirement is usually reduced, which results in lower O_2_ consumption and CO_2_ production (Ehrlich et al., 2016). In addition, processes to acquire nutrients are initiated that requires signaling cascades and membrane-bound proteins and enzymes (Campbell, 2007; He et al., 2009; McCue, 2010). Alterations in transcription due to nutritional changes is of interest since patient suffering from various diseases also have effects on metabolism, e.g., patients with anorexia (Heilbronn et al., 2007) and obesity (Barnes et al., 2011) as well as neurodegenerative diseases such as Alzheimer’s, Parkinson’s, and Huntington’s disease (Cai et al., 2012). The gene expression of *Unc93a* in mice has not, to our knowledge, been studied previously and mRNA was found in the central nervous system and peripheral tissues. The protein localization and the mRNA expression corresponded in the brain. Earlier, our group reported expression changes in *Unc93a* gene expression in N25/2 hypothalamic mouse cells subjected to amino acid starvation, where the mRNA was induced after the first 5 h and downregulated at 16 h (Hellsten et al., 2017b; Perland et al., 2017a). Building on this, we studied the gene expression alterations *in vitro* and *in vivo*. In mouse embryonic cortex cells subjected to amino acid deprivation, the same transient alteration was observed as in the N25/2 hypothalamic mouse cell line, where at 3 h *Unc93a* gene expression was upregulated, followed by a reduction in expression at 12 h. Furthermore, we studied *Unc93a* expression in mice subjected to complete starvation for 24 h, where we observed increased mRNA in peripheral tissues such as intestine and kidneys, both important for ADME of substrates, as well as lungs, thymus and heart. The effect in the central nervous system was limited to cerebellum, hypothalamus, olfactory bulb and spinal cord.

The main source of energy for nerve cells and the body as a whole is glucose, although, amino acids, especially essential amino acids that cannot be produced by the cell itself, are needed for metabolic processes and for cell-to-cell signaling (Mergenthaler et al., 2013; Hellsten et al., 2017b). Furthermore, a steady state of the ion balance is important to maintain to ensure proper cell signaling cascades, membrane potentials, enzyme activation and co-transport of different nutrients (Halperin and Kamel, 1998; Duprat et al., 2007; Pramod et al., 2013; Hellsten et al., 2017a). The mRNA expression changes observed from *Unc93a* does not follow other studied putative transporters, e.g., *Mfsd1*, *Mfsd3*, *Mfsd14a*, and *Mfsd14b* (Lekholm et al., 2017; Perland et al., 2017b). None of the transporters were upregulated by amino acid starvation in the hypothalamic cell cultures (Perland et al., 2017a), however, *Mfsd1*, *Mfsd14a*, and *Mfsd14b* was upregulated during amino acid deprivation in the primary cortex cells after 3 h, but they stayed upregulated (*Mfsd1*) or were not affected at all after 12 h (*Mfsd14a* and *Mfsd14b*). In mice subjected to starvation, similar transcript changes were observed between the studied putative transporters and *Unc93a* in some of the tissues such as upregulation in cerebellum and downregulation in hypothalamus (Lekholm et al., 2017; Perland et al., 2017b), although the peripheral organs has not been studied for the other putative transporters.

Amino acid starvation in the N25/2 mouse hypothalamic cell culture increased the expression of several genes important for different pathways in the cell. An early induction in genes involved in amino acid transport and transcription regulation are seen probably in an attempt to increase the uptake of the missing biomass (Efeyan et al., 2015; Hellsten et al., 2017b). The early induction on mRNA expression in the two *in vitro* experiments points to the possibility that *Unc93a* has an early role in the response to energy availability in these two types of cell cultures and maybe *Unc93a* has a role in sensing the nutrient limitation, energy reduction or amino acid uptake similar to the sodium dependent amino acid transporter *Slc38a2*. An early induction on transcript level during amino acid starvation is typically seen for *Slc38a2* (Palii et al., 2004; Tanaka et al., 2005; Hellsten et al., 2017b), which is known to be regulated by a set of transcription factors, such as ATF4 and C/EBP, that binds to the AAREs present in the first intron of the gene (Palii et al., 2004). ATF4 is a master regulator that is crucial for adaptation to stresses by regulating genes, involved in metabolism, nutrient uptake, antioxidant stress and apoptosis, by binding to the AARE (Kilberg et al., 2005; Dey et al., 2012). Similar C/EBP is a transcription regulator important for genes involved in metabolism, where C/EBPα has a more general role and C/EBPβ is more involved in fine-tuning responses (Ramji and Foka, 2002). When analyzing the promoter region for mouse *Unc93a*, both motifs for ATF4 and C/EBP were found. The *Unc93a* transcription level induction observed *in vitro* and *in vivo* are probably caused by the presence of ATF4 and C/EBP motifs in the promoter. However, the NSRU and the AAREs ASNS and CHOP could not be found in the promoter or *Unc93a* sequence obtained from the mouse genome. Furthermore, the mRNA induction for many amino acid transporters, for example *Slc38a2*, remains upregulated after a longer period of amino acid starvation and deprivation (Hellsten et al., 2017b), which is not seen for *Unc93a*, indicating that it is involved in alternative steps within the same pathway or another pathway. Other *in vivo* and *in vitro* studies have reported that *Slc2a1* (GLUT1) and *Slc2a3* (GLUT3) expression are induced after 72 and 48 h, respectively, of starvation in mammals (Wertheimer et al., 1991; Nagamatsu et al., 1994). The induction observed in the mouse brain for the *Slc2a3* was limited to cortex and hippocampus; two structures in which the transcript level of *Unc93a* were not affected after starvation. In addition, only two members of the SLC2 family were regulated by amino acid starvation in the hypothalamic cell culture, *Slc2a1* (downregulated) and *Slc2a12* (upregulated) during all time-points (Hellsten et al., 2017b), indicating that *Unc93a* is probably not involved in similar pathways as the SLC2 family.

ATF4 is not only a master regulator of genes involved in metabolic processes it is also essential for stress-induced autophagy, a process where macromolecules and organelles that are not needed are degraded to provide nutrients (Huett and Xavier, 2010; Benjamin et al., 2013; Tan et al., 2017). The increase in transcript levels of *Unc93a* observed *in vitro* and *in vivo* might indicate that it is involved in autophagy to provide the body with needed macronutrients during starvation, and it is possible that *Unc93a* is involved in the initiation of autophagy. Furthermore, the alterations observed for *Unc93a* expression in hypothalamus and olfactory bulb, two organs important for feeding (van den Top et al., 2004; Caba et al., 2014; Betley et al., 2015) further highlight its role in metabolism.

UNC93A has been reported to be expressed on the plasma membrane (Liu et al., 2002) and therefore one can assume that UNC93A can be important for sensing or transportation of nutrients and/or ions or, similar to UNC93B1, be involved in regulating and trafficking of membrane-bound proteins to, e.g., the plasma membrane to increase the membrane permeability for nutrients and/or ions. The same transient regulatory pattern seen for *Unc93a* in the hypothalamic cell culture from mice is also observed for other ion transporters (Montalbetti et al., 2013; Hellsten et al., 2017b), especially the iron transporter *Slc40a1*. The fact that *Unc93a*is regulated in a similar fashion as *Slc40a1* (Hellsten et al., 2017b)and is needed together with the TASK channels in *C. elegans* to regulate ion flow in the muscles (de la Cruz et al., 2003), make it possible that UNC93A is important to keep the ion levels steady in the cell during normal conditions and challenges, e.g., it might regulate ion inflow important for cellular pH, antioxidant stress responses and osmolality either directly or indirectly by regulating other transporters.

A multicellular model system does not react to nutrient scarcity as a unicellular system since homeostatic responses aimed to maintain the circulating nutrient levels are available as well as storage capacity (Efeyan et al., 2015; Ehrlich et al., 2016). The transient alteration, early upregulation and later downregulation, observed in cell cultures and the late upregulation observed in adult mice can be due to the differences in the setup of starvation on amino acids vs. complete food starvation. It could also possibly be due to the fact that an animal have other processes, storage and organs contributing to energy homeostasis during starvation, which the hypothalamic cell line and primary cortex cultures lack. However, all of the results point to *Unc93a* having a vital and general function in cellular metabolism, either by regulating the nutrient, end-product or stress level. However, it is difficult to pin-point the exact role of UNC93A in these processes that occurs during different nutrient availabilities. In adult mice the intentional experiment was to monitor changes due to nutrient availability, but as other parameters such as stress and immune responses were not measured, the gene regulation seen could be due to other confounding factors and not just energy metabolism. It is therefore possible that the induction of *Unc93a* is caused by stress and immune responses, which can be further strengthened since UNC93A has ATF4 motifs present in its promoter, a transcription factor reacting to integrated stress responses, UNC93A has been reported to be important for viral assembly in mosquitoes (Campbell et al., 2011) and its closest related protein, UNC93B1, is highly involved in the innate immunity (Lee et al., 2013; Huh et al., 2014).

## Conclusion

(I) UNC93A is a protein with 12 transmembrane helices, that fold similar to other MFS and (II) UNC93A is expressed in brain and peripheral tissues. (III) *Unc93a* has ATF4 and C/EBP motifs present in the promoter region which probably regulate its transcription during nutritional challenges. (IV) The response to amino acid availability *in vitro* indicates a role in sensing nutrient limitation, changes in energy, autophagy or transportation of nutrients. (V) The similar transcript change observed between *Unc93a* and *Slc40a1* also point to the possibility that *Unc93a* is important for ion regulation during cellular challenges such as starvation and/or stress. (VI) Taken together Unc93a seem to have a vital and general role in energy homeostasis and cellular metabolism. Even though the function and the substrate profile of UNC93A are still unclear, the specific histological expression of the transporter both in central nervous system and peripheral tissues makes this transporter interesting to study further. The gene regulation results observed for *Unc93a* both *in vitro* and *in vivo*, due to nutrient availability, is a reason to perform more research as it is clearly involved in regulation and/or sensing of important cellular pathways and mechanisms, providing a clear example why transporters are important to study further.

## Author Contributions

MC: Designed experiments, prepared material for qRT-pcr and performed immunohistochemistry, immunocytochemistry, qRT-PCR, and analysis, set up primary cultures, imaging, complied figures and tables and drafted the manuscript. EL: Set up starvation experiments and primary cultures, performed WB on HEK293 samples, aided in analysis of results, imaging of fluorescent immunohistochemistry on sections and primary cultures, and wrote parts of the manuscript. SH: planned and performed deprivation experiments in primary cell cultures, prepared tissue, performed non-fluorescent DAB IHC, imaging and complied figure for DAB, wrote parts of the material and methods, assisted in analysis of qRT-PCR and assisted in WB. EP: Set up starvation experiments, performed proteomic analysis, complied tables and wrote parts of the section “Material and Methods.” RF: Designed the project, performed proteomic analysis and aided in data analysis and drafted the manuscript. All authors have read and approved of the manuscript and helped with interpretation of results.

## Conflict of Interest Statement

The authors declare that the research was conducted in the absence of any commercial or financial relationships that could be construed as a potential conflict of interest.

## Footnotes

1 http://www.jmol.org/

2 https://www.ncbi.nlm.nih.gov/homologene

3 http://www.phylomedb.org/

## Supplementary Material

**FIGURE S1 |** Verification and protein localization of a N-terminus anti-UNC93A antibody. There are other antibodies on the market for UNC93A and the N-terminal antibody (ab173552, Abcam) was also verified by Western blot on a protein homogenate from mouse brain. Two concentration were tested and staining with N-terminal UNC93A gave two bands at approximately 52 and 35 kDa in **(A)** the supernatant (S2) but not the pellet (P1). **(B)** The N-terminal UNC93A (lower raw) was also compared to the UNC93A (ab69443) used in this paper (upper raw). The two staining’s corresponded, but the N-terminal UNC93A was more nuclear. The N-terminal antibody was not further used in the protein localization study. UNC93A is in green and the nuclear marker DAPI is blue. Scale bars represent 20 μm.
