# Supplementary figures and images for: The Neuronal and Peripheral Expressed Membrane-Bound UNC93A Respond to Nutrient Availability in Mice
Source: Front Mol Neurosci. 2017 Oct 31;10:351. doi: 10.3389/fnmol.2017.00351 (PMC5671512; doi:10.3389/fnmol.2017.00351)

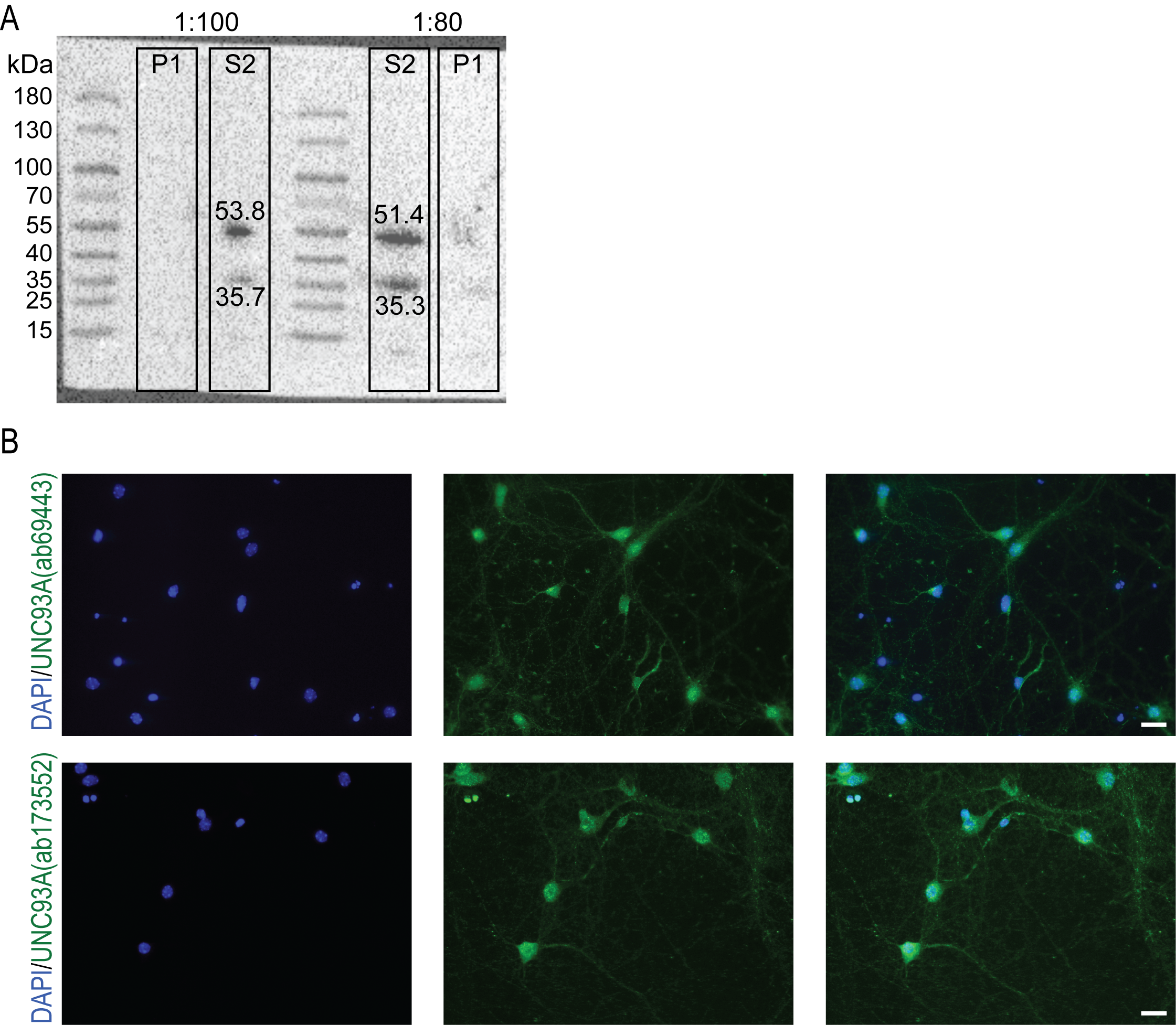

Supplement: Supplementary file 1 [file Image_1.TIF]
